# Knowledge, attitudes and perceptions of occupational hazards and safety practices in Nigerian healthcare workers

**DOI:** 10.1186/s13104-016-1880-2

**Published:** 2016-02-06

**Authors:** Olufemi Oludare Aluko, Ayobami Emmanuel Adebayo, Titilayo Florence Adebisi, Mathew Kolawole Ewegbemi, Abiodun Tolani Abidoye, Bukola Faith Popoola

**Affiliations:** Department of Community Health, Faculty of Clinical Sciences, Obafemi Awolowo University, Ife, Nigeria

**Keywords:** Workplace hazards, Hazards in healthcare facilities, Healthcare workers, Nigeria

## Abstract

**Background:**

By profession, healthcare workers (HCWs) attend to clients and patients through a variety of preventive and curative services. However, while their attention is focused on providing care, they are vulnerable to hazards that could be detrimental to their health and well-being. This is especially true in developing countries where health service delivery is fraught with minimal protective precautions against exposures to numerous fomites and infectious agents. This study assessed the workplace hazards and safety practices by selected HCWs in a typical health care facility (HCF) in Nigeria.

**Methods:**

The study utilized a descriptive cross-sectional design and stratified sampling technique to identify 290 respondents. The study used mixed methodology and collected data by validated instruments with resulting data analyzed by IBM-SPSS, version 20.

**Results:**

The results showed that over half of the respondents were registered nurses, female, married (61.7 %) with 5 years median work experience (70.3 %). Most respondents (89 %) were knowledgeable about hazards in HCFs, identified recapping used needles as a risky practice (70 %) and recognized that effective hand washing prior to, and after every clinical procedure in preventing cross infection (100 %). Also, most respondents (96.2 %) believed they were at risk of occupational hazards while about two-thirds perceived the risk as high. In addition, only 64.2 and 87.2 % had completed Hepatitis B and Tetanus immunizations, respectively. Only 52.1 % “always” complied with standard procedures and most (93.8 %) practice safe disposal of sharps (93.8 %) while those that did not (40 %) generally implicated lack of basic safety equipment. In this study, the practice of hand washing by respondents was not influenced by occupation and education.

**Conclusions:**

The high level of knowledge demonstrated by respondents was at variance with practice, therefore, measures aimed at promoting safety practices and, minimizing exposure to hazards such as; provision of safety equipment, pre-placement and routine training of staff on safety practices and adequate reinforcement of staff capacity and capability through drills in all HCFs should be institutionalized and made mandatory. The protocol of the safety training and drills should be responsive to evidence-based emerging and sectoral safety challenges.

**Electronic supplementary material:**

The online version of this article (doi:10.1186/s13104-016-1880-2) contains supplementary material, which is available to authorized users.

## Background

Health care facilities (HCFs) are institutions that provide health care services, including counseling, clinical, surgical, and/or psychiatric consultations and treatment services for the healthy, sick and the injured [1]. Globally, HCFs employ over 59 million workers and offer variety of services to clients and patients, and are classified as hazardous and high risk work place [2, 3]. Healthcare facilities like other high risk work places are characterized by a high level of exposure to hazardous agents, which significantly endangers the health and life of workers (HCWs).

Hazards are an inherent property of a substance, agent, source of energy or situation that has the potential of causing undesirable consequences while risk is the probability that damage to ‘life, health, and or the environment’ may occur from a hazard. In this regard, occupational hazards refer to workplace activities that have the potential to cause/increase the risk of injury or ill health [4–6]. Occupational safety is the control of hazards in the work place to achieve an acceptable level of risk, while workplace safety generally refers to the process of protecting the health and safety of staff while on the job, irrespective of vocation [6, 7].

Occupational health and safety is an important issue because of high rates of associated morbidity and mortality of exposed workers. An estimated 100,000 people die from occupational illnesses, while about 400,000 new cases of occupational diseases are diagnosed every year [8, 9]. This affects workers in various occupations as a result of their exposure to different types and varying degrees of hazards in the workplace. However, studies indicate that workers in the farming, general contracting, steel, automobile, truck driving and nursing sectors have the highest risk of exposure to high risk occupational hazards [9].

According to Oluwagbemi [7], HCFs in Nigeria have increased in magnitude, sophistication and diversity over the last 30 years with challenges on ensuring and sustaining best practices and equipment required to perform high risk clinical procedures. However for HCFs, protecting the health and well being of front line health care workers (HCWs) is difficult [7]. In discharging their statutory duties, HCWs may be exposed to hazards which significantly impair their health and quality of life, with multiplier effect on their immediate and extended family members. Thus, HCWs need protection from workplace hazards as much as the staff in other high risk workplaces such as mining or construction works.

The hazards in HCFs are classified by WHO (2002) into physical, biological, mechanical, ergonomic, chemical and psycho-social. Previous studies have shown that occupational injuries and illnesses among HCWs ranked among the highest of any industry though could be reduced or eliminated. The predominant hazards to HCWs include blood-borne infections [Human Immunodeficiency Virus (HIV), Hepatitis B virus (HBV) and Hepatitis C virus (HCV)], back and neck pain, burn-out stress, allergic reactions to latex materials, spills from chemicals, exposure to radiation, assault from patients; among others [10]. The factors that contribute to occupational illnesses and injuries in HCFs include negligence and carelessness of health care workers, lack of adequate protective aids and equipment, inadequate number of staff, excessive workload, failure to observe basic safety and hygiene guidelines, and inadequate operational knowledge of modern healthcare equipment [10]. These prompted the US Centre for Disease Control and Prevention (CDC) to develop standard precautions (SPs) for preventing occupational exposures and handling of infectious materials in HCFs [11, 12]. Adherence to the SP guidelines has been shown to be effective in curtailing occupational illnesses and injuries among HCWs in HCFs [13].

The consequences of occupational ilnesses and injuries include physical, economic and psychological damages to the HCWs and their dependants [14]. In Nigeria, HCWs (medical doctors, nurses and nursing assistants) are poorly prepared to handle occupational hazards and therefore sustain injuries/illnesses while performing their duties [15]. The vulnerability of staff in the HCFs is compounded by the inadequacy of facilities with equipment that could enhance best practice in developing countries.

Occupational vulnerability of HCWs therefore threaten the quality health care delivery in developing countries, especially among Medical Doctors, Nurses and Nursing Assistants. A higher percentage of the few studies on occupational hazards among HCWs in developing countries focused on specific job designations within the healthcare delivery system. Whereas, this study assessed the occupational hazards and safety practices among the healthcare personnel whose job descriptions entail having direct contacts with patients on an almost daily basis in majority of the HCFs—the Medical Doctors, Nurses and Nursing Assistant (MdNNA). In this regard, this study identified the determinants of occupational hazards and predisposing factors among the selected HCWs in the study location in Nigeria.

## Methods

### Description of the study location

**Fig. 1:**
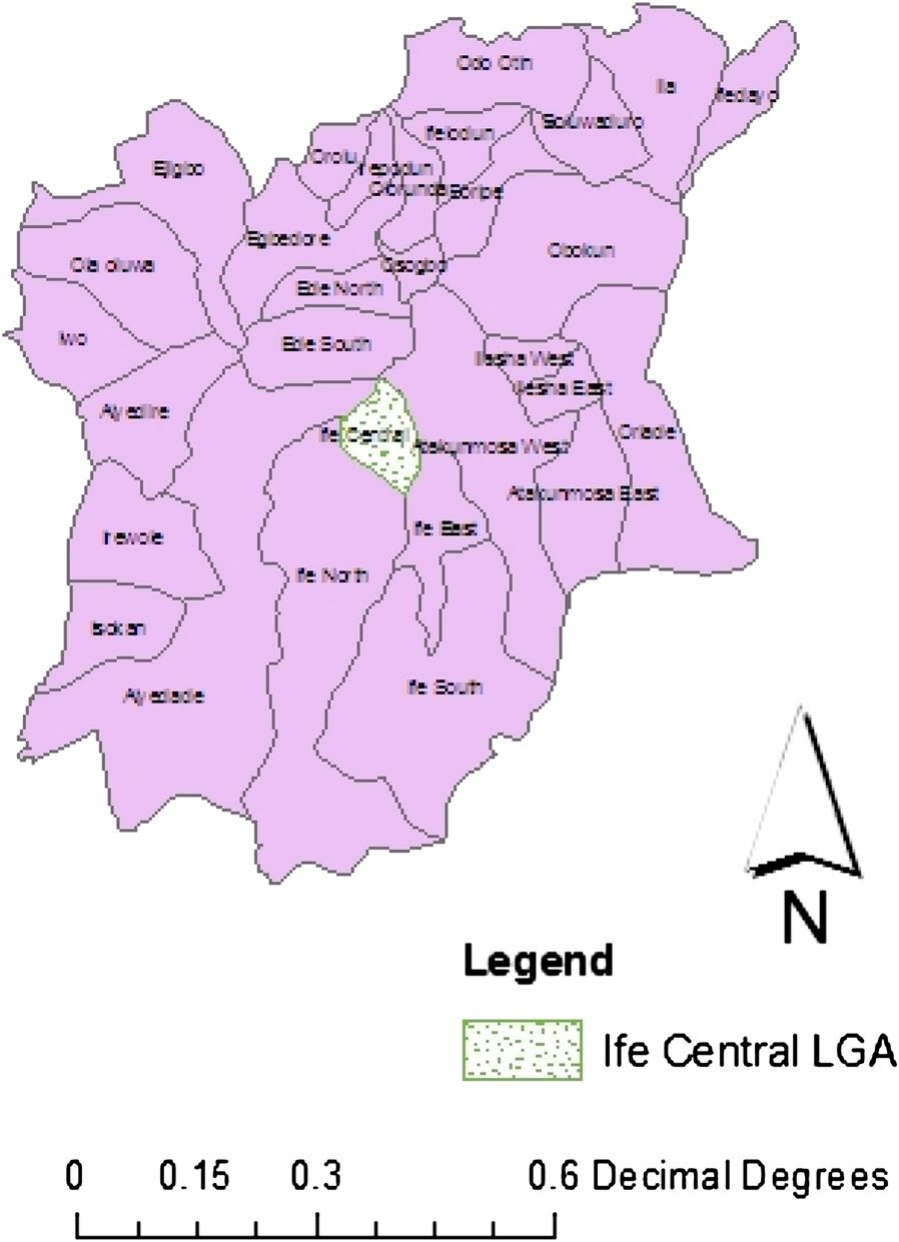
Map of Osun State showing the location of the study area

The study was conducted in Osun State, one of the 36 States in Nigeria (Fig. 1). Osun State occupies 9250 km^2^ approximately and has over 200 towns. The climate is essentially tropical, with humid, high temperatures, and distinct wet and dry seasons. As at 2006, the State is inhabited by 3,416,959 people, comprised of 1,734,149 (50.8 %) males and 1,682,810 (49.2 %) females [16]. The study took place in Ile Ife, the headquarters of Ife Central LGA and one of the 776 LGAs in Nigeria. The LGA is bounded by Ife North, Ife East, Ife South and Atakunmosa West LGAs. The HCF, where the study took place has 26 wards and is a Federal Government-owned, tertiary healthcare facility. The hospital has in its services medical doctors, nurses, nursing assistants, laboratory scientists, dentists, medical records officers, physiotherapists, administrators, pharmacists and environmental health officers, among others though the study target population were Medical Doctors, Nurses and Nursing Assistants (MdNNAs). In fulfilling its mandate, the HCF focuses on, an integrated healthcare delivery approach with emphasis on broad and comprehensive healthcare service to promote improvement in the physical, mental and socio-economic well-being of Nigerians through preventive, promotive, diagnostic, restorative and rehabilitative services.

The HCF has undergone strategic changes in administration, management, physical resources and services since its establishment though its philosophy on provision of comprehensive health care services; based on integrated, primary, secondary and tertiary health care delivery remained constant. The HCF has 342 bed complement for patients and has in-patients admissions (257,981); consultant out-patients (1,653,455); General out-patients (2,335,684) while accident/emergency and deliveries records were 571,604 and 64,029 since 1977 [17].

### Study design

The study utilized a descriptive cross-sectional design. The sampling frame, from the hospital records comprised all Medical Doctors (410), Nurses (547) and Nursing Assistants (53) while those randomly selected (313) through proportional to size sampling technique were the study population.

### Sample size determination

The sample size was determined using Fisher’s formula for estimating single proportions [18] and the formula for estimating the minimum sample size when the total sample was below 10,000 people [19]. The standard normal deviation was set at a 95 % confidence level, prevalence at 64.6 % [20] with the allowable margin of error of 5 %. The Fisher’s formula (n = Z^2^(pq)/d^2^) gave a sample size of 350 respondents which was reduced to 261 when the formula [18] (ns = n/(1 + n/N) was applied since the total eligible respondents was (1010) and less than 10,000 respondents. In addition, the minimum sample size increased to 286, and was rounded up to 290 when 10 % of the calculated, minimum sample size was added for non-response, inappropriately filled or missing questionnaires since the questionnaires were interviewer administered due to educational background of some nursing assistants. In the two formulae:

- n = minimum required sample size in population greater than 10,000
- ns = minimum required sample size in population less than 10,000
- Z = Standard normal variate for 95 % confidence level, (Z = 1.96)
- p = prevalence of the attribute (64.6 %)
- d = acceptable difference; using 5 % (d = 0.05)
- q = 1 − p
- N = population size.

### Sampling technique

The study utilized a stratified sampling technique. The total number of respondents in the three category of HCWs was obtained through a proportional sampling to size method. The summation of the three categories of HCWs (1,010) was used to calculate proportions for Medical doctors (118), Nurses (157) and nursing assistants (15), respectively. Moreover, the respondents were stratified into wards based on their representativeness (spread), with simple random sampling used to identify respondents among various sections in the 26 wards in the HCF.

### Data collection and analysis

Our study took place between February and March 2014. The study utilized a modified version of the validated data collection questionnaire [13] (Additional file 1) and Focus Group Discussion (FGD) (Additional file 2). The study instruments were pretested at the University Health Centre of the Obafemi Awolowo University. Errors and ambiguous questions sequencing were corrected prior to field data collection. The study instruments had sections on socio-demographic information, knowledge, attitudes and practices on occupational exposures, risk and history of occupational injury and prophylaxis after exposure to health compromising conditions in the HCF by respondents. The study questionnaire was interviewer-administered in the 26 wards. Of the 313 respondents, only 302 consented while 290 returned filled instruments that were used in the study; a response rate of 92.7 %.

The data collected were checked and cleaned by the researchers for completeness and accuracy. The questionnaires were coded, entered, and analyzed using IBM-SPSS version 20. The data in the entered questionnaires were tested for internal consistency and had very high Cronbach’s α value of 0.850, which indicates a high level of internal consistency for our scale that comprised 116 study items. Moreover, socio demographic characteristics and other univariate variables were presented by summary statistics using mean, median and standard deviation for continuous variables and frequency/percentages for categorical variables.

In addition, we used express scribe software for verbatim transcription of the audio recordings of the FGDs, followed by primary and secondary coding that identified themes relevant to the study which were, abstracted and used in supporting and triangulating the study findings.

Furthermore, the questionnaire respectively had 13, 17 and 8 questions on knowledge, attitude and perception of respondents. Prior to analysis, positive responses were coded as ‘1’ while negative responses were coded ‘0’ for knowledge and perception questions. Attitude was measured by 5 point Likert scale: “Strongly agree”, “agree”, “undecided”, “disagree” and “strongly disagree” and scored 5, 4, 3, 2 and 1, respectively. For knowledge, attitude and perception variables, cumulative scores were aggregated and categorized into high/good and low/poor (knowledge and perception); while attitude was categorized as positive and negative, respectively.

In addition, the median (inter-quartile range) for attained knowledge score were 11 (10–12). So, a score below 11 was considered low and a score equal to or greater than 11 was high [21]. Similarly, a score of 78 (74–80) and 6 (5–7) were used to stratify the composite scores of attitude and perception on what constitute occupational hazards in the workplace into positive and negative, good and poor categories, respectively.

For bivariate analysis, Chi-square statistics was used to assess the type of relationship between professions of HCWs and other categorical variables and selected occupational hazards indices by the Pearson Chi-square statistics, except otherwise stated, with level of significance at <5 %.

### Summation of KAP scores into category

The scores by individual respondent in knowledge and perception scales were stratified into high and low; good and poor categories while attitude scores was stratified into positive and negative categories. The scores of each of the respondents was assessed by considering their linear composite scores in knowledge, attitude and practice (KAP) scales together with high, positive and good scores repressented by 1; while low, negative and poor scores were represented by 2, respectively. Similar trends in KAP were aggregated into 8 possible groups as follows: high, positive, good (1,1,1) scores in KAP was represented by 1; low, negative, poor (2,2,2) scores in KAP was represented by 2; high, positive, poor (1,1,2) scores in KAP was represented by 3; high, negative, poor (1,2,2) scores in KAP was represented by 4; high, negative, good (1,2,1) scores in KAP was represented by 5; low, positive, good (2,1,1) scores in KAP was represented by 6; low, negative, good (2,2,1) scores in KAP was represented by 7 while low, positive, poor (2,1,2) scores in KAP was represented by 8, respectively (Additional file 3).

In addition, the frequency of respondents that were represented by 1, 2, 3, 4, 5, 6. 7 and 8 were aggregated. All those that had good ratings in knowledge, attitude and practice were rated as “good KAP” and those that had poor scores in the three scales were rated as “poor KAP” In-between categories showed possible associations in the study population (Fig. 4).

### Inclusion and exclusion criteria

This study was limited to only 26 out of the 28 wards in the hospital because of the difficulty in assessing and engaging healthcare workers in the surgical and the intensive care wards of the hospital. In addition, due to the nature of the study, administrative healthcare workers, irrespective of their professions were excluded from the study.

### Ethical approval

The study protocol (IPH/OAU/12/229) was approved by the Ethical Committee of the Institute of Public Health of the Obafemi Awolowo University in Nigeria. Prior to respondents’ verbal consent, they were informed about the purpose of the study and were made to understand that participation was voluntary and refusal to participate in the study attracts no penalty. The study respondents were assured of confidentiality and personal identifiers were removed in the summary data to ensure confidentiality. Though the questionnaires were distributed to randomly selected respondents in the 26 wards, it bears only codes and administered at various times and shift work duties to guarantee confidentiality. In addition, only the research team members had access to electronic data, encrypted and stored only in the external hard drive of the principal investigator. Besides, the data collection was anonymous and participants in the FGDs were randomly picked and borne identification codes only.

## Results

### Socio-demographic characteristics of respondents

The socio-demographic characteristics of respondents are presented in Table 1. The study revealed that over half (152) respondents were nurses while 55.5 % were females. The mean age (SD) of respondents was 33.4 ± 7.4 years, while 234 (80.7 %) were between 20 and 39 years. In this study, most respondents were married (61.7 %), Christians (93.8 %) and belonged to Yoruba ethnicity (86.9 %). Moreover, the respondents’ median duration of working experience was 5 years, though over half (52.8 %) had worked for between 1 and 5 years. In addition, most respondents (70.3 %) had Bachelor degree qualifications (Table 1).

**Table 1:** Socio-demographic characteristics of respondents

Description of variables	Frequency (n)	Percentage (%)
*Age range of respondents (n* *=* *290)*		
≤19	02	0.7
20–29	100	34.5
30–39	134	46.2
40–49	39	13.4
50–59	15	5.2
*Sex (n* *=* *290)*		
Male	129	44.5
Female	161	55.5
*Religion (n* *=* *290)*		
Christianity	272	93.8
Islam	16	5.5
Traditional	02	0.7
*Ethnicity (n* *=* *290)*		
Yoruba	252	86.9
Igbo	37	12.8
Hausa	01	0.3
*Marital status (n* *=* *290)*		
Single	110	37.9
Married	179	61.7
Others (divorced)	01	0.3
*Occupation (n* *=* *290)*		
Medical doctor	123	42.4
Nurse/midwife	152	52.4
Nursing assistant (ward maid)	15	5.2
*Years of working experience (n* *=* *290)*		
1–5	153	52.8
6–10	78	26.9
11–15	29	10.0
16–20	10	3.4
>20	20	6.9
*Educational qualification (n* *=* *290)*		
Secondary school leaving certificate	06	2.1
Nursing diploma certificate	61	21.0
Bachelor’s degree	204	70.3
Completed master’s degree	19	6.6

### Knowledge of respondents on occupational hazards in health care facilities

**Fig. 2:**
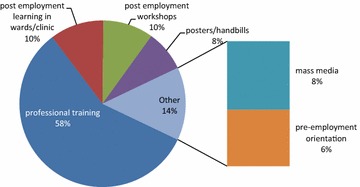
Sources of knowledge on occupational hazards by respondents

Table 2 showed the knowledge of respondents on occupational hazards and safety. Eighty-nine percent (258) of respondents had knowledge on the possible hazards in the HCFs while 70 % (202) respondents had knowledge that recapping used needles after use negates the recommendation in the standard precaution guidelines. In addition, all respondents knew that hand washing is essential to preventing cross infection after clinical procedures (Table 2). On composite knowledge index, the study showed that 167 respondents (57.6 %) had high knowledge while 123 (42.4 %) respondents had low knowledge on occupational hazards and safety in the workplace. Furthermore, most (58 %, 253) respondents acquired knowledge on occupational hazards through professional training while only 6 % respondents acquired it through pre-employment orientation on work ethics (Fig. 2).

**Table 2:** Knowledge of respondents on occupational hazards and safety practices

Description of knowledge on occupational hazards	High knowledge level	Low knowledge level
	n (%)	n (%)
Awareness about occupational hazards in the health care facility	289 (99.7 %)	1 (0.3 %)
Knowledgeable on occupational hazards and their categories	258 (89 %)	32 (11 %)
Knowledgeable on occupational infections.	250 (86.2 %)	40 (13.8 %)
The most likely sources of occupational infections	250 (86.2 %)	40 (13.8 %)
Procedures where needle stick injuries are most likely to occur	266 (91.7 %)	24 (8.3 %)
Procedures that violate the standard precautions	202 (69.7 %)	88 (30.3 %)
Occupational cross infection after clinical procedures could be prevented by effective hand washing	290 (100 %)	0.0 (0.0 %)
Knowledgeable on physical hazards	238 (82.1 %)	52 (17.9 %)
Knowledgeable on chemical hazards	237 (81.7 %)	53 (18.3 %)
Knowledgeable on biological hazards	210 (72.4 %)	80 (27.6 %)
Knowledgeable on ergonomic hazards	98 (33.8 %)	192 (66.2 %)
Knowledgeable on mechanical hazards	185 (63.8 %)	105 (36.2 %)

### Attitude of respondents towards hazards and safety practices

In our study, 288 (99.4 %) respondents believed that occupational hazards should be prioritized and promptly considered as part of conducive work provisions in healthcare facilities and 285 (98.3 %) believed that prevention and control of hazards in the healthcare facilities should be a shared responsibility between the hospital management and staff. In addition, 287 (99 %) perceived staff training and provision of protective equipment as mandatory to reduce their risk of exposure to occupational hazard while 287 (99 %) emphasized reporting and documentation of all exposures to occupational hazards by appropriate authorities. Also, 234 (81 %) respondents believed that strict punitive sanctions should be measured out on staff members that violate safety practices in the healthcare facility to deter others (Table 3). On composite attitude index, our study showed that 232 respondents, constituting 80 % had positive attitude in contrast to 20 % that had negative attitude towards occupational hazards and preventive safety practices.

**Table 3:** Attitude of respondents towards occupational hazards and safety practices

Perception on occupational hazards	Agree	Undecided	Disagree
	No. (%)	No. (%)	No. (%)
Occupational hazard should be taken seriously and given prompt attention in the hospital	288 (99.4 %)	0 (0.0 %)	2 (0.6 %)
Prevention of occupational hazard is a joint responsibility of the hospital management and the staff	285 (98.3 %)	1 (0.3 %)	4 (1.4 %)
Paying extra attention to occupational hazard is an unnecessary burden on me	36 (12.5 %)	12 (4.1 %)	242 (80.4 %)
Training of staff and provision of personal protective equipment is necessary to reduce the risk of exposure to occupational hazard	287 (99 %)	0 (0.0 %)	3 (1.0 %)
Aprons and face masks should be worn in procedures where splash/spill of blood is likely	288 (99.3 %)	0 (0.0 %)	2 (0.7 %)
Gloves should always be worn when administering injections, starting IVs and drawing blood	286 (98.6 %)	2 (0.7 %)	2 (0.7 %)
Hands should be properly washed after each contact with a patient	287 (99.0 %)	2 (0.7 %)	1 (0.3 %)
Used needles should NEVER be recapped	235 (81.1 %)	7 (2.4 %)	48 (16.5 %)
Sharps should be disposed in sharps’ boxes	285 (98.2 %)	3 (1.0 %)	2 (0.7 %)
Safety boxes should be located at close distances to where required procedures are administered	278 (95.8 %)	4 (1.4 %)	8 (2.7 %)
HBV, Measles, Mumps, Rubella and Influenza vaccines should be received by all health workers	275 (94.8 %)	7 (2.4 %)	8 (2.7 %)
Prolonged standing by HCWs should be avoided	256 (88.3 %)	22 (7.6 %)	12 (4.1 %)
All exposures to occupational hazards should be reported and documented by appropriate authorities	287 (99.0 %)	2 (0.7 %)	1 (0.3 %)
Adequate staffing of HCFs will reduce occupational hazards	214 (93.0 %)	10 (3.4 %)	10 (3.5 %)
Incentives should be provided for adhering to universal standard precautions	243 (83.8 %)	34 (11.7 %)	13 (4.5 %)
Punitive actions should be taken against HCWs that violates standard safety precautions and practices	234 (81.0 %)	41 (14.0 %)	15 (5.0 %)
Exposure and infection control policies (standard operating procedures) should be regularly reviewed and updated by the hospital management	288 (99.0 %)	2 (1.0 %)	0 (0.0 %)

### Awareness on occupational hazards and safety practices by respondents

**Fig. 3:**
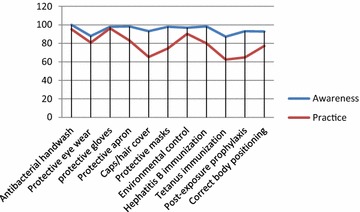
Awareness and practice on safety precautions by respondents

As shown in Table 2, our study showed that most respondents (99.7 %) were aware about occupational hazards and respectively recognized physical (82 %), chemical (81.7 %), biological (72.4 %), mechanical (63.8 %) and ergonomic (33.8 %) hazards. Also, most respondents (289, 99.7 %) were aware of safety precautions against occupational hazards. In addition, 194 (66.9 %) respondents were aware about job aids for their professions. As presented in Fig. 3, our study revealed that 98.3 % and 87.2 % among the respondents were respectively aware about complete immunization against Hepatitis B virus and Tetanus while 93.1 % knew about post-exposure prophylaxis. In the study, wearing hand gloves for routine clinical procedure is practiced by 279 (96.2 %) respondents. Moreover, 242 (77.2 %) respondents practice correct body positioning during clinical procedures while 272 (93.8 %) respondents practice safe disposal of injection needles and sharps. However, only 181 (62.4 %) respondents have been completely immunized against Hepatitis B virus (Fig. 3).

On compliance with preventive procedures in the HCF, only 52.1 % respondents reported to ‘always’ comply with standard occupational safety precautions. However, the 47.9 % that do not ‘always’ comply implicated lack of safety kits/equipment (41.0 %), compliance waste time (5.9 %) and made them uncomfortable (1.0 %) as reasons.

### Perception on occupational hazards

Our study revealed potential risk of infections (e.g. from needle prick injury) and musculoskeletal problems (e.g. low back pain) from prolonged standing and stress related conditions due to strenuous job demands, consequent of inadequate staffing in the HCF. However, the responsibility for preventive practices was majorly on staff that, according to the study; ‘*they bear the brunt and consequences of exposure to occupational hazards*’ In addition, an FGD participant said “*they* (*health care workers) had the tendency to keep quiet due to minimal expectations from the hospital management in cases of occupational injuries*” and therefore undermine completeness of reported cases of job-related injuries in the HCF.

The above report was confirmed by quantitative data, which showed that 279 (96.2 %) respondents believed that they are at risk of exposure to occupational hazards in the hospital, and about two-third (174 respondents) perceived the risk of exposure to be high while 31.7 % and 8.6 % considered the risk as medium and low, respectively. The staff perceived susceptibility from needle prick injuries (94.5 %), direct contact with patients’ fluid (92.4 %) and assaults from patients (77.2 %) as major hazards, while routine night shifts (59.3 %) and body contact with patients having HIV (52.1 %) were least considered as hazards (Table 4). Also, more than two-third respondents (67.2 %) perceived their vulnerability to occupational hazards to be high while 95 (32.8 %) felt that their work practices do not compromise their health, sufficiently.

**Table 4:** Perceived risk of occupational hazards

Types of occupational hazards	Yes		No	
	Frequency	Percent	Frequency	Percent
Needle prick injury	274	94.5	16	5.5
Body contact with retroviral patients	151	52.1	139	47.9
Exposure to radiation	219	75.5	71	24.5
Assault from patients	224	77.2	66	22.8
Assault from co-workers	184	63.4	106	36.6
Direct contact with patients’ body fluid	268	92.4	22	7.6
Routine night shifts	172	59.3	114	39.3

### Composite rating scores of knowledge, attitude and practice

**Fig. 4:**
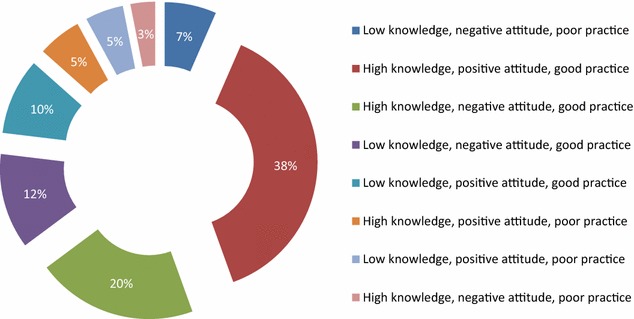
Composite knowledge, attitude and perception of occupational hazards among health care workers

The composite KAP of respondents revealed that close to two-fifth (38 %) had positive rating in KAP while, one-fifth (20 %) had poor knowledge, good attitude and perception. In addition, 12 percent respondents showed poor knowledge, good attitude and poor perception while one-tenth (10 %) showed good knowledge and attitude but poor perception. The study further showed that only 7 % respondents had negative rating in the three scales (Fig. 4).

### Measure of association among socio-demographic indices and occupational hazards variables

Our study revealed statistical significant associations between occupational categories and knowledge (χ^2^ = 32.236, P = 0.000) and attitude (χ^2^ = 14.194; P = 0.001) while there was no significant difference between work categories and perception on vulnerability to occupational hazards (χ^2^ = 3.614; P = 0.164) (Table 5). Also, and work experience was significantly associated with occupational categories (P = 0.000); education was significantly associated with knowledge (P = 0.000) while marital status was associated with respondents’ perception (0.039). In addition, ethnicity was associated with attitude (P = 0.028) while religion was associated with perception (P = 0.013) and attitude (P = 0.016). Furthermore,, sex was significantly associated with knowledge (P = 0.20) and attitude (P = 0.007), as shown in Table 6.

**Table 5:** Association between knowledge, attitudes and perceptions and occupations of respondents

	Knowledge			Attitude			Perception		
	Low	High	Total	Negative	Positive	Total	Poor	Good	Total
*Occupations*									
Medical doctor	39 (31.7 %)	84 (68.3 %)	123 (42.4 %)	37 (30.1 %)	86 (69.9 %)	123 (42.4 %)	64 (52.0 %)	59 (48.0 %)	123 (42.4 %)
Nurse	69 (45.4 %)	83 (54.6 %)	152 (54.6 %)	18 (11.8 %)	134 (88.2 %)	152 (52.4 %)	92 (60.5 %)	60 (39.5 %)	152 (52.4 %)
Nursing Assistant	15 (100 %)	0 (0 %)	15 (5.2 %)	3 (20.0 %)	12 (80.0 %)	15 (5.2 %)	11 (73.3 %)	4 (26.7 %)	15 (5.2 %)
Total	123 (42.4 %)	167 (57.6 %)	290 (100 %)	58 (20.0 %)	232 (80.0 %)	290 (100.0 %)	167 (57.6 %)	123 (42.4 %)	290 (100 %)
	Chi-square, Likelihood Ratio (χ_LR_^2^) = 32.236; degree of freedom (df) = 2; P value (P) = 0.000. (Significant)			χ_LR_^2^ = 14.194; df = 2; P = 0.001. (Significant)			χ_LR_^2^ = 3.614; df = 2; P = 0.164. (Non-significant association)

**Table 6:** Association among selected occupational hazards and socio-demographic variables

Education\knowledge	Poor	Good	Total
Diploma	36 (59.0 %)	25 (41.0 %)	61 (21.0 %)
Bachelors’ degree	72 (35.5 %)	132 (64.7 %)	204 (70.3 %)
Masters’ degree	9 (47.4 %)	10 (52.6 %)	19 (6.6 %)
Others (secondary school certificate)	6 (100.0 %)	0 (0.0 %)	6 (2.1 %)
Total	123 (42.2 %)	167 (57.6 %)	290 (100.0 %)
Chi-square, likelihood ratio (χ^2^_LR_) = 21.574; degree of freedom (df) = 3; P value (P) = 0.000 (significant)			

Marital status\perception	Poor	Good	Total
Single	27 (24.5 %)	83 (75.5 %)	110 (37.9 %)
Married	68 (38.0 %)	111 (62.0 %)	179 (61.7 %)
Divorced	0 (0.0 %)	1 (100.0 %)	1 (0.3 %)
Total	95 (32.8 %)	195 (67.2 %)	290 (100.0 %)
χ^2^_LR_ = 6.506; df = 2; P = 0.039 (significant)			

Ethnicity\attitude	Negative	Positive	Total
Yoruba	45 (17.9 %)	207 (82.1 %)	252 (86.9 %)
Igbo	12 (32.4 %)	2 (67.6 %)5	37 (12.8 %)
Others	1 (100.0 %)	0 (0.0 %)	1 (0.3 %)
Total	58 (20.0 %)	232 (80.0 %)	290 (100.0 %)
χ^2^_LR_ = 7.539; df = 2; P = 0.006 (significant)			

Religion\perception	Poor	Good	Total
Christianity	94 (34.6 %)	178 (65.4 %)	272 (93.8 %)
Islam	1 (6.2 %)	15 (93.8 %)	16 (5.5 %)
Traditional	0 (0.0 %)	2 (100.0 %)	2 (0.7 %)
Total	95 (32.8 %)	195 (67.2 %)	290 (100.0 %)
χ^2^_LR_ = 8.641; df = 2; P = 0.013 (significant)			

Occupations\work experience	1–5 years	>5 years	Total
Medical doctor	80 (65.0 %)	43 (35.0 %)	123 (42.4 %)
Nurse	62 (40.8 %)	90 (59.2 %)	152 (52.4 %)
Nursing assistant	11 (73.3 %)	4 (26.7 %)	15 (5.2 %)
Total	153 (52.8 %)	137 (47.2 %)	290 (100 %)
χ^2^_LR_ = 19.006; df = 2; P = 0.000 (significant)			

Religion\attitude	Negative	Positive	Total
Christianity	58 (21.3 %)	214 (78.7 %)	272 (93.8 %)
Islam	0 (0.0 %)	16 (100.0 %)	16 (5.5 %)
Traditional	0 (0.0 %)	2 (100.0 %)	2 (0.7 %)
Total	58 (20.0 %)	232 (80.0 %)	290 (100.0 %)
χ^2^_LR_ = 8.236; df = 2; P = 0.016 (significant)			

The study further showed that occupation was not significantly associated with respondents’ perception of occupational hazards (P = 0.68) and handwashing (0.295); education was not significantly associated with hand washing (P = 0.455) and perception (P = 0.239). In like manner, religion was not significantly associated with knowledge (P = 0.206) and attitude (P = 0.130); marital status was not associated with handwashing (P = 0.887) and knowledge (P = 0.12) while sex was not statistically associated with perception (P = 0.283) (Table 6).

## Discussion

Health care facilities are work places where nosocomial infections predominate and diseases pathogens are haboured by fomites and pests. The situation could be aggravated by absence of appropriate protective measures, excessive workload, inadequate training of workers on safety practices, among others, especially in developing countries. Healthcare workers are prone to occupational hazards, injuries and diseases. The situation in a tertiary HCF, which should be seen and emulated as an example of good practices in the health care industry was studied to understand the magnitude of its occupation hazards and safety-related challenges.

### Socio-demographic characteristics of respondents

In our study, most respondents were nurses, females and married, in consonance with previous study findings [22]. The mean age of respondents was 33 years; lower than 44 years reported by Manyele et al. [22] in a similar study in Tanzania. In addition, most respondents belonged to Yoruba ethnicity (86.9 %), probably due to the study location, the cradle of Yorubaland, though a Federal government HCF in contrast to the study of Ford and Tetrick [5] where ethnicity of respondents vary widely; from white (55 %), Black (28 %), Asian (12 %) and other races (5 %). Moreover, most of the respondents had less than 5 years work experience and had Bachelor’s degree qualifications. However, the work experience of respondents in this study is similar to that reported by Ford and Tetrick [5].

### Knowledge of respondents on occupational hazards and preventive practices

Knowledge, according to concise oxford dictionary is information and skills, acquired through experience and/or education. Knowledge of potential occupational hazards and safety in HCFs is germane to forming positive attitude that will inform behaviour. In this regard, most respondents were aware about types of hazards and hazardous situations in the 26 wards studied, except ergonomic hazards. Ergonomic hazard doesn’t appear to be well recognized as an hazard in the HCF and this was also observed in a study in Namibia where only 38 % of the respondents were aware of ergonomic hazards [23].

In our study, most respondents were aware that recapping used needles is a risky practice that predisposes HCWs to occupational hazards. Similarly, all respondents knew that effective hand washing after each and every clinical procedure is essential in preventing cross infection. The observation by Oluwagbemi [7] that HCWs are exposed to a variety of hazards that might differ according to education or job description was evident from our study where occupational differences and different job duties exposed HCWs to different hazards. According to our study, the knowledge differentiation by occupation of HCWs showed that all nursing assistants had poor knowledge in agreement with the findings by Ford and Tetrick [5] and Tziaferi et al. [6].

In contrast to the findings by Anisha [24] and Viragi et al. [25], over half respondents in our study had high knowledge rating. However, Nurses and Dentists only were respondents in Anisha [24] and Viragi et al. [25] studies while our study focused on MdNNAs. However, knowledge differential was evident when the respondents were categorised by professions. Hence, all nursing assistants had poor knowledge despite admission, through FGD to pre-employment orientation and regular workshops, where they were orientated on handwashing, the use of latex gloves and safe handling and disposal of sharps. Furthermore, the sources of knowledge on occupational preparedness to identify hazards and indulge in safety practices were majorly acquired through in-school professional training while very few HCWs had it through pre-employment orientation on work ethics. The above was in agreement with previous observations, which indicated that the pre-employment orientation modules and mode of delivery should be strategically reviewed to strengthen positive staff awareness on occupational safety and the use of standard operation procedures by HCWs.

The commonest source of knowledge of occupational hazards identified by our study was in-school professional training (87.2 %), on the job experience, and post-employment professional in-service workshops. The above was at variance with the findings in similar studies in Belgaum city in India and Ile Ife in Nigeria, where most respondents acquired knowledge on occupational hazards and safety practices from post-employment seminars [25, 26].

### Awareness on occupational hazards and safety practices by respondents

Evidenced from our study, HCWs were aware of, and recognized variety of occupational hazards, such as physical hazards (82 %), chemical hazards (81.7 %), biological hazards (72.4 %), mechanical hazards (63.8 %) and ergonomic hazards (33.8 %). Most of them (99 %) were aware about preventive safety precautions, mostly through job aids (66.9 %). In addition, most respondents were aware of full immunization against Hepatitis B virus (98.3 %), Tetanus (87.2 %) and post-exposure prophylaxis (93.1 %) and knew that they are at risk of exposure to occupational hazards, in agreement with findings by Fasunloro and Owotade [26] in Ile Ife, Nigeria, and Amosu et al. [10] in Abeokuta, Nigeria where most respondents knew that their vocation predispose them to occupational hazards.

### Attitude towards occupational hazards and safety practices by respondents

The summation of attitude revealed that most respondents (80 %) had positive attitude towards safety practices and prevention of occupational hazards. This could be due to high knowledge showed by respondents and as a result of fear of occupational infections and illnesses which could be terminal and life threatening in some instances. Again, many HCWs attend in-service training workshops that have sessions on safety precautions in HCFss. They believed that this should be a shared responsibility between hospital management (with the infection control committee playing a major role) and the staff, who bear the consequences of occupational injuries with their dependants.

Handwashing is a widely recognized process of preventing cross infection in HCFs. In this study, the attitude of respondents to handwashing was positive. This observation is good since HCWs give their best to patients while paying little attention to their own health, especially in emergency situations [27]. In addition, almost all participants (99 %) considered capacity building on occupational hazards and safety, documentation of occupational exposures as well as post exposure prophylaxis to reduce the risk and effect of exposures. Generally, personal protective equipment (PPE) shields HCWs from occupational hazards in clinical procedures. Hence, HCWs should be sanctioned in hospitals where PPE were provided but not used, though in most cases, the personal safety devices were non-existent. Moreover, some respondents believed that observing safety precautions against occupational hazards can be relatively burdensome and time-consuming, though wearing of aprons and gloves are considered to be very important before clinical procedures, and needles are not to be recapped.

### Practice on occupational hazards and safety practices by respondents

Healthcare workers experience exposure to numerous occupational hazards due to the unique nature of their work [28]. Hence the preventive coping strategies were examined in this study. Our study revealed that wearing hand gloves for clinical procedures was practiced by most (96.2 %) respondents while an improvement is sought by those (22.8 %) that practice incorrect body positioning during clinical procedures. In addition, sharps and injection needles were safely disposed (93.8 %) while all HCWs should be fully immunized against Hepatitis B virus where only 62.4 % respondents had it as at the time of the study. Surprisingly, only 52.1 % “always” complied with preventive safety precautions advised in the standard operating procedures (SOPs) and job aids. However, the reasons given by defaulting 47.9 % that do not ‘always’ comply with SOPs were lack of, or inadequacy of safety kits/equipment (41.0 %), time compliance (5.9 %) and associated discomfort (1.0 %).

### Perception of occupational hazards

The risk of exposure was high in our study since most respondents (96.2 %) are at risk of exposure to occupational hazards and about 2/3 respondents perceived the risk of exposure as high. This was in agreement with findings of previous studies by Orji et al. [15], Manyele et al. [22], Amosun et al. [10], Tziaferi et al. [6], Chiou et al. [28] and; Enwere and Diwe [20]. The three mostly reported occupational exposures among the respondents were needle prick injuries (94.5 %), direct contact with patients’ body fluid (92.4 %) and assault from patient (77.2 %) while the potential outcomes of occupational hazards were risk of infection (e.g. from needle prick injury), musculoskeletal problems (e.g. low back pain) and stress related conditions arising from intense job demands, consequent of inadequate staffing. The above findings were in agreement with previous findings where psychological distress, burnout, absenteeism, employee intent to leave, reduced patient satisfaction, and diagnosis and treatment errors were consequent of occupational hazards [29]. However, the responsibility for preventive practices were mostly placed on HCWs that, according to a participant in one of the FGDs, ‘*bear the brunt and consequences of exposure to occupational hazards*’ In addition, an FGD participant said “*health care workers had the tendency to keep quiet due to minimal expectations from the hospital management*’ and therefore undermine the reported cases of occupational injuries in the hospital environment. Therefore, efforts should be made to neutralise stigma associated with HCWs that reported exposures to occupational hazards and immediate access post exposure prophylaxis.

### Comparative analogy of knowledge, attitude and practice of respondents

According to WHO (2012), the rational model of health promotion believed high knowledge, will translate to positive attitude and subsequently good behaviour, though in reality, the transition is not straight forward but depended on several factors [30]. In this regard, the study compared the distribution of respondents by performance on composite knowledge, attitude and practice (KAP). In our study, about two-fifth (38 %) had positive rating in KAP while 7 % had negative rating in KAP scales. However, more than half were in various categories that required interventions to reduce occupational hazards in the HCF.

On relationship among variables, the respondents’ occupation and sex categories were significantly associated with knowledge and attitude while education was also associated with knowledge, in agreement with the findings of Tziaferi et al. [6] where it was concluded that the level of education influences the level of knowledge in health and safety issues [6]. This meant that those with high knowledge had better education, in agreement with the rational model [30]. In like manner, marital status and religion of respondents influence practice while ethnicity, sex and religion influence their attitude. To engender safety precautions that will decimate the prevalence of occupation hazards in the HCF, attention should be focused on knowledge based awareness creation disseminated around marriage relationship and religion, to achieve desired results. Conversely, respondents practices were not influenced by sex and education while knowledge on occupational hazards and safety practice was not influenced by marital status and religion. Also, hand washing practice was not influenced by education and marital status. The above findings were in agreement with Tziaferi et al. [6] where the authors concluded that level of education and professional specialty influenced their perception of risk level at a statistically significant level.

### Study limitations

The study was cross sectional in design and could not establish a causal relationship among study variables [31]. The study findings is also prone to respondents’ bias arising from the use of semi-structured questionnaire, though the data obtained was triangulated with those of FGD interviews. The study was conducted in a tertiary healthcare facility, a model of best practice in the health care sector and so; the findings might not be generalisable across levels of HCFs in Nigeria since situations elsewhere might be worse than presented in this study.

## Conclusions

The flaws identified by this study has far and wide consequences on the health of workers in the HCF, and consequently on the quality of healthcare delivered to patients. Therefore, this required a concerted efforts by all stakeholders, especially the hospital infection control committee, the management of the HCF, as well as government to adequately neutralise work related hazards accross the healthcare industry in Nigeria. Therefore, adequate provision of appropriate safety kits, their timely replacement when worn out and updated job aids should be made available to all cadres of staff based on their job schedule. Through policy, pre-employment and routine training and safety precautionary drills should be institutionalised for health care workers on occupational hazards and safety practices. In addition, full immunization of all HCWs against vacine preventable, contagious diseases, timely documentation of all cases of exposure to occupational hazards. In addition, the post exposure prophylaxis unit in hospitals should be strenghtened for immediate response to manage the aftermaths of exposure of HCWs to occupational hazards. Besides, enforcement of existing laws on occupational safety at work places should be monitored to increase adherence by public and private healthcare providers. This should also include strategic policy level measures to increase personnel shortages in the HCFs towards achieving the personnel:patient ratio in all HCFs.

Furthermore, health promotion and routine monitoring visits should paid to sections in HCFs, especially by the infection control committee and the committee should also have the wherewithal that will prolong the life and longevity of service by HCWs in our HCFs in Nigeria.

## Additional files

10.1186/s13104-016-1880-2 Validated questionnaire on the knowledge, attitude and perception among Dcotors, Nurses and Nursing assistants on occupational hazards in a tertiary healthcare facility in Nigeria.
10.1186/s13104-016-1880-2 Focus group discussion guide.
10.1186/s13104-016-1880-2 Summation of composite knowledge, attitude and perception ratings.

## Abbreviations

CDC: US Centre for Disease Control and Prevention
d: acceptable difference; using 5 % (d = 0.05)
df: degree of freedom
FGD: Focus Group Discussion
HBV: Hepatitis B virus
HCFs: health care facilities
HCV: hepatitis C virus
HCWs: health care workers
HIV: human immunodeficiency virus
IBM-SPSS: international business machine-statistical packages for social sciences
KAP: knowledge, attitude and practice
LGA: local givernment area
MdNNAs: medical doctors, nurses and nursing assistants
n: minimum required sample size in population greater than 10,000
N: population size
ns: minimum required sample size in population less than 10,000
p: prevalence of the attribute
P: P value
PPE: personal protective equipment
SD: standard deviation
SOP: standard operating procedure
SP: standard precaution
WHO: World Health Organisation
χ: Chi-square
Z: Standard normal variate for 95 % confidence level
