# Supplementary material for: Knowledge, attitudes and perceptions of occupational hazards and safety practices in Nigerian healthcare workers
Source: BMC Res Notes. 2016 Feb 6;9:71. doi: 10.1186/s13104-016-1880-2 (PMC4744628; doi:10.1186/s13104-016-1880-2)
Supplement: Supplementary file 1 — 10.1186/s13104-016-1880-2 Validated questionnaire on the knowledge, attitude and perception among Dcotors, Nurses and Nursing assistants on occupational hazards in a tertiary healthcare facility in Nigeria. [file 13104_2016_1880_MOESM1_ESM.docx]

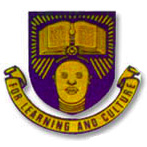
**ANNEX I**

**QUESTIONNAIRE ON THE KNOWLEDGE, ATTITUDE AND PERCEPTION AMONG DOCTORS AND NURSES AND NURSING ASSISTANTS ON OCCUPATIONAL HAZARDS IN A TERTIARY HAELTH CARE FACILITY IN NIGERIA.**

| **Introduction/Informed consent:**  Sir/Ma, my name is ______________. I thank you for agreeing to be part of this study. You have been randomly selected to provide knowledge, experiences and perception on wide and varied issues on occupational hazards and challenges being faced in this hospital. This questionnaire was designed solely for the purpose of research by a group of research faculty and students at the Obafemi Awolowo University, Ile-Ife. The Research will enable us to understand the situation and identify ways forward on how to efficiently improve the current situation. The time spent on the instrument should be about 45 minutes.  All responses will be kept confidential. This means that your responses will only be shared with research team members for the purpose of this study only and we will ensure that any information we include in our report does not identify you as the respondent. Your participation is completely voluntary and there is no penalty for refusing to participate. We solicit your cooperation in completing all relevant sections of this questionnaire. We thank you for your anticipated cooperation.  **DECISION: 1. Respondent agrees to be interviewed [ ] 2. Respondent did not agre to be interviewed [ ].**  **If 2, thank the respondent, end the interview and move to the next selected respondent.** |
| --- |

**SECTION A**: (**SOCIO – DEMOGRAPHIC DATA OF RESPONDENTS)**

1. Age (as at last birthday)___________________
2. Sex: 1. Male[ ] 2.Female [ ]
3. Religion:1. Christianity [ ] 2. Islam [ ] 3. Traditional [ ] 4. Others [ ]
4. Ethnicity: 1.Yoruba [ ] 2. Igbo [ ] 3. Hausa [ ] 4. Others [ ]
5. Marital Status: 1. Single [ ] 2.Married [ ] 3. Divorced[ ] 4. Separated [ ] 5. Widow/widower [ ]
6. Occupation: 1. Doctor [ ] 2. Nurse [ ] 3. Nursing Assistant [ ]
7. Current Department:
8. Years of working experience
9. Highest educational qualification: 1. No formal education [ ] 2. Diploma [ ] 3.Bachelors’ degree [ ] 4. Masters’ Degree [ ] 5. Others (specify)

**SECTION B: (KNOWLEDGE, AWARENESS AND PERCEPTION)**

**Instruction:** *please, tick the appropriate options*

1. Do you know about occupational hazards? 1. Yes[ ] 2. No[ ]
2. Which one of the following is **NOT** an occupational hazard in this hospital?

1. Noise[ ] 2. Needle stick injuries[ ] 3. Early arrival at work[ ] 4.Body contamination with patients’ body fluids[ ]

1. Which one of the following is **NOT** an occupational infection in this hospital?1. HBV[ ] 2. HIV[ ] 3. Chicken pox[ ] 4. Malaria[ ]
2. The **MOST** likely source of occupational infections is one of the following:(a) air-borne (b) faeces and urine (c) blood and body fluids (d) body contact
3. During which of the following activities is a needle stick injury **MOST** likely to occur? (a) recapping (b) transporting to the sharp’s disposal safety box (c) handling equipment before use (d) handling equipment after disposal
4. Which of the following violates the Standard Precautions? (a) aspirating for blood before intramuscular injections (b) recapping needles after use (c) leaving needles attached to syringes after use
5. Hand washing is good to prevent occupational cross infection after procedures(a) Yes [ ] (b) No[ ] (c) Don’t know [ ]
6. What type(s) of occupational hazards are you aware of? *(you can tick more than one)* 1. Physical[ ] 2. Chemical[ ] 3 .Biological[ ] 4. Ergonomic[ ] 5. Mechanical[ ]
7. **Awareness/practice of safety precautions**
8. Are you aware of safety precautions against occupational hazards? 1. Yes [ ] 2. No [ ]

If yes, which of the following precautions are you aware of and which do you practice?

| **Precautions** | **Awareness** | | **Use/Practice** | |
| --- | --- | --- | --- | --- |
|  | **Yes** | **No** | **Yes** | **No** |
| 1. 19. Hand washing with bactericidal agent |  |  |  |  |
| 20 Barrier methods: |  |  |  |  |
| 21. Gloves |  |  |  |  |
| 22. Gowns (apron) |  |  |  |  |
| 23. Caps |  |  |  |  |
| 24. Masks (goggles) |  |  |  |  |
| 25. Environmental control e.g. effective waste handling |  |  |  |  |
| 26. Safe disposal of sharps |  |  |  |  |
| 27. Complete immunization against:   1. Hepatitis B 2. Tetanus |  |  |  |  |
| 28. Prophylactic treatment and/or procedures following exposures |  |  |  |  |
| 29. Correct body posture during procedures |  |  |  |  |

1. Do you have job aids (e.g. instructions) stating procedure and safety precautions on your job? 1. Yes ( ) 2. No ( )

31. How often do you comply with safety precautions?

1. Always 2. Often 3. Sometimes 4.Never

32. If **NOT** “always”, why? 1. Makes me feel uncomfortable [ ] 2.Compliance wastes time [ ] 3. Unavailability of safety kits [ ]

33. Why is it important to comply with safety precautions? 1. Because it’s the hospital policy [ ]. 2. For personal and patients’ safety [ ] 3. Others (Please specify):________________

1. **Perception of occupational hazards**
2. Do you think you are at risk of occupational hazards 1.Yes[ ] 2. No[ ]

35. If yes, to what degree1. High[ ] 2.Medium[ ] 3. Low[ ]

Do the following constitute occupational hazards to you? *Please tick as appropriate.*

| **Description** | 1. **YES** | 1. **NO** |
| --- | --- | --- |
| 36. Needle Prick |  |  |
| 37. Body contact with retroviral positive patients |  |  |
| 38. Exposure to radiation |  |  |
| 39. Assault from patient |  |  |
| 40. Direct contact with patient’s body fluid |  |  |
| 41. Assault from co-workers |  |  |
| 42. Recapping of needle after use |  |  |
| 43. Weekly night shifts |  |  |

**SECTION C: (ATTITUDE TOWARDS HAZARDS/SAFETY PRACTICES) INSTRUCTION:** *Please, tick as appropriate*

| **DIRECTIONS** | **Strongly agree** | **Agree** | **Undecided** | **Disagree** | **Strongly disagree** |
| --- | --- | --- | --- | --- | --- |
| 44. Occupational hazard is an issue that should be taken seriously and given prompt attention in the hospital |  |  |  |  |  |
| 45. Prevention of occupational hazards is a joint responsibility of the hospital management and the staff |  |  |  |  |  |
| 46. Paying extra attention to occupational hazard is an unnecessary burden on me? |  |  |  |  |  |
| 47. Training of staff and provision of personal protective equipment is necessary to reduce the risk of exposure to occupational hazard |  |  |  |  |  |
| 48. Aprons and face masks should be worn in procedures where splash/spill of blood is likely |  |  |  |  |  |
| 49. Gloves should always be worn when administering injections, starting IVs and drawing blood |  |  |  |  |  |
| 50. Hands should be properly washed after each contact with a patient |  |  |  |  |  |
| 51. Used needles should NEVER be recapped |  |  |  |  |  |
| 52. Sharps should be disposed in sharps’ boxes |  |  |  |  |  |
| 53. Disposal boxes should be located within a few feet of where you practice |  |  |  |  |  |
| 54. HBV, Measles, Mumps, Rubella and Influenza vaccines should be received by all health workers |  |  |  |  |  |
| 55. Prolonged standing should be avoided by all health workers |  |  |  |  |  |
| 56. All exposures to occupational hazards should be reported to and appropriately documented by appropriate authorities |  |  |  |  |  |
| 57. Adequate staffing of hospitals is a way of reducing occupational hazards |  |  |  |  |  |
| 58. There should be provision of incentives for adherence to universal safety precautions |  |  |  |  |  |
| 59. Punitive actions should be taken against violators of safety practices |  |  |  |  |  |
| 60. Exposure and Control policies should be regularly reviewed by the hospital management |  |  |  |  |  |

**SECTION D: (RISK AND PREDISPOSING FACTORS)**

**1.Risk**

How many times have you had occupational hazard conditions in the LAST TWO MONTHS? (p*lease tick as appropriate).*

| **Hazard** | **Once** | **Two times** | **Three times** | **More than three times** | **Never** |
| --- | --- | --- | --- | --- | --- |
| 61. Needle pricks |  |  |  |  |  |
| 62. Latex Allergies |  |  |  |  |  |
| 63. Constant exposure to radiation |  |  |  |  |  |
| 64. Direct contact with body fluids(blood, urine, etc) |  |  |  |  |  |
| 65. Trips, slips and falls |  |  |  |  |  |
| 66. Heavy lifting e.g. patients, equipment |  |  |  |  |  |
| 67. Chemical spill |  |  |  |  |  |
| 68. Assaults from patient |  |  |  |  |  |
| 69. Assaults from co workers |  |  |  |  |  |
| 70. Assaults from patient’s relative |  |  |  |  |  |
| 71. Work overload |  |  |  |  |  |
| 72. Sleepless nights |  |  |  |  |  |
| 73. Poorly ventilated working environment |  |  |  |  |  |
| 74. Anaesthetic gases |  |  |  |  |  |
| 75. Fire outbreak |  |  |  |  |  |

76. When was the last time that you had exposure to an occupational hazard in this hospital? 1. Within the last two months( ) 2. Within two to six months( ) 3. Within six to 12 months ( )

4. > 1 year ( )

77. When you were exposed, did you report the last incidence to the appropriate section in the hospital? 1. Yes ( ) 2. No ( ) [*If No, skip 80 and 81*]

78. Did you receive any post-exposure treatment? 1. Yes ( ) 2. No ( ) [*If No, skip 81*]

79. Were you satisfied with the post exposure treatment? 1. Yes ( ) 2. No ( )

**II. Predisposing factors for occupational hazards in your workplace**

**INSTRUCTION: (***Please tick as appropriate)*

| **Factors** | **Agree** | **Disagree** | **Not sure** |
| --- | --- | --- | --- |
| 80. Inadequate hand washing facility |  |  |  |
| 81. Lack of awareness about safety practices in health care settings |  |  |  |
| 82. Lack of commitment on the part of management to invest in infection control programs |  |  |  |
| 83. Individuals negligence and carelessness |  |  |  |
| 84. Lack of adequate protective aids and equipment |  |  |  |
| 85. Shortage of staff |  |  |  |
| 86. Prolonged standing |  |  |  |
| 87. Inadequate knowledge of usage of modern facilities |  |  |  |

**SECTION E: (MEASURES AGAINST OCCUPATIONAL HAZARDS)**

**INSTRUCTION: (***Please tick as appropriate)*

***NB:*** *HCWM - Health Care Waste Management*

88. Where did you get your information on occupational hazards? 1. During my training [ ] 2. Post-employment professional workshop [ ] 3. Ward rounds/Clinics[ ] 4. pre-employment orientation [ ] 5.Through posters and handbills in the hospital [ ] 6. Media[ ] 7.. Others (Please, specify) ________________

89. Do you consider the information adequate? 1. Yes[ ] 2. No[ ]

90. Have you been trained on occupational hazards and prevention strategies?

1. Yes, on injection safety only ( ) 2. Yes, on HCWM only [ ] 3. Yes, on injection safety and HCWM [ ] 4.On safety practices in hospitals[ ] 5. No ( )

91. How many times have you attended trainings on occupational hazard exposure and preventive strategies in the last 12 months? 1. Once ( ) 2. Twice( ) 3. Thrice( ) 4. More than three times ( ) 5. None ( )

92. Have you heard/seen any communication on various types of occupational hazards in the last one month? 1. Yes [ ] 2. No [ ]

93. Which of these channels do you prefer for messages about occupational hazard messages? 1. TV [ ] 2. Radio [ ] 3.Posters [ ] 4.Professional meetings [ ] 5.Others (Pls specify)___________

94. Do you know about any protocols that are in place to deal with occupational hazards in this hospital? 1. Yes( ) 2. No( )

95. If Yes, mention those protocols:

____________________________________________________________________________________________________________________________________________________________

96. In this hospital, is there a designated unit that manages staff occupational hazard and exposure? 1. Yes and operational( ) 2. Yes but not operational( ) 3.No( )

97. Are you on any health insurance? 1. Yes ( ) 2. No ( )

98. If yes, do you consider its coverage adequate? 1. Yes( ) 2.No ( )
