# Supplementary material for: Knowledge, attitudes and perceptions of occupational hazards and safety practices in Nigerian healthcare workers
Source: BMC Res Notes. 2016 Feb 6;9:71. doi: 10.1186/s13104-016-1880-2 (PMC4744628; doi:10.1186/s13104-016-1880-2)
Supplement: Supplementary file 2 — 10.1186/s13104-016-1880-2 Focus group discussion guide. [file 13104_2016_1880_MOESM2_ESM.docx]

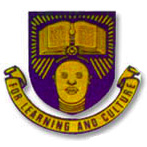


**KNOWLEDGE, PERCEPTION AND PRACTICE AMONG DOCTORS AND NURSES AND NURSING ASSISTANTS ON OCCUPATIONAL HAZARDS IN A TERTIARY TEACHING HOSPITAL IN NIGERIA**

**FOCUS GROUP DISCUSSION GUIDE**

**Introduction**

I want to thank you all for taking the time to meet with us today. Our names are --------------------------------------- and we would like to talk to you about your knowledge and attitudes towards your occupation, occupation related hazards and preventive practices in this hospital. We will be asking you questions to understand your perceptions, thoughts and experiences to help us understand the situation and challenges around the subject matter.

The FGD should take about 90 minutes. We would like to tape the session because we don’t want to miss any of your comments. __________ will be taking some notes during the session, but he/she can’t possibly write fast enough to get it all down. Because we’re on tape, please speak up so that we don’t miss your comments.

All responses will be kept confidential. This means that your interview responses will only be shared with research team members for the purpose of this study and we will ensure that any information we include in our report does not identify you as the respondent. Members of the research team will be the only people that listen to the recording of our conversation today. The only time we will share information with others outside of our team is if we think your safety or the safety of others is in danger. Remember, you don’t have to talk about anything you don’t want to and you may end the interview at any time. Your participation is completely voluntary and there is no penalty for refusing to participate.

**Can we continue the discussion? Yes ( ) No ( )**

**GROUND RULES**

Before we start, we would like to set some ground rules. It is very important that whatever we discuss remains here. We want everyone to feel comfortable sharing their experiences with the group. We know that many of you have experiences that you are interested in sharing, and we want to make sure we can hear from each and every one of you. Let’s respect our fellow participants and when they speak, do not interrupt but let them finish first and then speak your mind. Also sometimes you might have a different opinion or different experience from someone else in the group. It’s important to us to hear all kinds of different opinions and experiences, so please do not feel shy if you have something different to say, and please also respect what other people in the group say.

One thing we would like to add: Sometime you may have questions that you are shy with sharing with the group. It would be very helpful for us to know what these questions are as we develop ways to help girls. We will give you pieces of paper to write these questions and we will give you a moment at the end to please write any questions. We will not be able to answer them now, but they will help us in the future.

**Do you agree to these rules**?

We would like you to pick a discussion identification number for the purpose of being here. We will use this identification number throughout the course of the discussion.

**Is there anything you will want us to add?**

**Do you have any question for us before we begin**?

**Icebreakers**

- Can you please describe your daily routine, from the time you arrive in the hospital, until you go home at the end of your duty for the day.
- What is your favourite soup and why?

**QUESTIONS**

1. What is your opinion on occupational hazards vis-a-vis your job as a health care provider?
2. What are the hazards you have been exposed and the common hazards observed in the course of your work experience?

Please, expatiate on these...

1. What are the major factors that predispose you to hazards in the discharge of your duty? Please, expatiate on these…
2. What is your opinion on strict adherence to safety guidelines by health care providers?
3. What are the hospital arrangements and provisions to minimize exposure of staff to occupational hazards in this hospital?
4. Please, assess in-depth the post exposure prophylaxis arrangement in this hospital?.
5. What are the various practical means through which exposure of colleagues to occupational hazards could be prevented

- *Are there any other issues you want us to discuss that we did not mention?*
- *Do you have any other comments or questions?*
- *Please, do remember not to share our discussion with people when you go outside.*

*Thank you for your participation. Your involvement is really important to us, and we appreciate you sharing your ideas.*
